# Current Insights in Microbiome Shifts in Sjogren’s Syndrome and Possible Therapeutic Interventions

**DOI:** 10.3389/fimmu.2018.01106

**Published:** 2018-05-24

**Authors:** Christina Tsigalou, Elisavet Stavropoulou, Eugenia Bezirtzoglou

**Affiliations:** ^1^Laboratory of Microbiology, Medical School, Democritus University of Thrace, Alexandroupolis, Greece; ^2^Service de Médecine Interne, Centre Hospitalier Universitaire Vaudois (CHUV), Lausanne, Switzerland; ^3^Department of Agricultural Development, Democritus University of Thrace, Orestiada, Greece

**Keywords:** Sjogren’s syndrome, microbiome, dysbiosis, probiotics, prebiotics, therapeutic microbiota manipulations

## Abstract

Sjogren’s syndrome (SS) is an autoimmune disease, among the most common ones, that targets mainly the exocrine glands as well as extra-glandular epithelial tissues. Their lymphocytic infiltration leads to manifestations from other organs (e.g., kidneys, lungs, liver, or thyroid), apart from sicca symptoms (xerostomia and keratoconjunctivitis). SS is more prevalent in women than in men (9:1). Moreover, p.SS patients are in increased risk to develop lymphoma. Certain autoantibodies (e.g., antibodies against ribonucleoprotein autoantigens Ro-SSA and La-SSB) are ultimate hallmarks for the disease. It was not known until recently that culture-independent techniques like next-generation sequencing (NGS) facilitate the study of the microbe communities in humans and scientists achieved to define the outlines of the microbiome contribution in health and disease. Researchers have started to investigate the alterations in diversity of the oral, ocular, or intestinal microbiota in SS. Recent studies indicate that dysbiosis may play a significant role in SS pathogenesis. At the same time, the cause or effect is not clear yet because the dysfunction of salivary glands induces alterations in oral and intestinal microbiome which is linked to worsen of symptoms and disease severity. If the human microbiome proves to play a key role in pathogenesis and manifestation of SS, the next step could be new and promising therapeutic approaches such as probiotics or prebiotics. This mini review focuses on the alterations of microbiome of SS patients, their connection with immune tolerance and new therapeutic strategies involving diet manipulation toward future personalized medicine.

## Key Messages

- Sjogren’s syndrome is a multifactorial, autoimmune disease still underdiagnosed and undertreated.
- All of its pathophysiology and etiology aspects are not fully elucidated.
- Recent studies have strive to examine the potential interactions with the human microbiome as regards to all the disease aspects, exploiting vital information from other autoimmune conditions, namely, IBD, T1DM, etc.
- Diet interventions and supplementations for SS patients need to be thoroughly examined and researched to identify novel therapies and prevention measures in the context of personalized medicine.

## Introduction

Sjogren’s syndrome (SS) is an autoimmune disease, among the most common ones, possibly as common as rheumatoid arthritis (RA) that targets mainly the exocrine glands as well as extra-glandular epithelial tissues. Lymphocytic infiltration of salivary and lacrimal glands leads to dysfunction that is becoming obvious by the emergence of “sicca syndrome” (dry eyes–dry mouth). In addition, clinical symptoms could derive from autoimmune invasion (so called “autoimmune epithelitis”) (1) in other organs, namely, kidneys, lungs, etc. It is a heterogenous and multifactorial disease involving genetic, environmental, and hormonal parameters that may occur in any age with a male/female ratio 1:10 (2). SS is considered a benign condition although is characterized by high incidence of lymphoma. Unfortunately, it is a disease with poor diagnosis and undertreatment although the last decades a lot of efforts have been made by clinicians and researchers. The gap in therapeutic options may be filled by the recent advances in microbiome research. Dysbiosis has already been a suspect for SS’s pathogenesis like other inflammatory diseases and the scientific community examines now the possible interplay between human microbiome and SS clinical manifestations and severity. Mouse models of the disease have been implicated a lot during the last decades although the multifactorial origin of pSS poses certain cons to their usage. non-obese diabetic (NOD) mice manifest not only type I diabetes (T1D) but SjS-like autoimmune endocrinopathy as well and also present a few hallmark autoantibodies like ANA, anti-SSA/Ro, anti-SSB/La, anti-a-fodrin, etc. (3–5).

Several experimental approaches demonstrated that there is a link between gut microbiota alterations and disease manifestations, severity and treatment responsiveness. For example, it is now known that gut dysbiosis with a low relative richness of symbionts and high relative richness of pathobionts deteriorates SS-like disease in mouse model and correlates with SS severity in humans (6).

Interventions targeting restoration of the microbiota shift using for example food supplements is currently under the microscope to elucidate the effects on clinical status. Prebiotics and probiotics are considered modern strategies for gut microbiota modulation, and we herewith provide a mini review focused on the alterations of microbiome of SS patients, their microbiome–host interactions and novel treatments involving diet manipulation toward future personalized medicine.

## Short Digest in the Current Study of SS

Sjogren’s syndrome a heterogenous and multifactorial disease involving genetic, environmental, and hormonal parameters that may occur in any age with a male/female ratio 1:10 (2). When SS exists solely is called primary SS to make the distinction from the situation of secondary SS when another autoimmune disease coexist or preexisted, namely, systematic lupus erythematosus or RA. In the lymphocytic infiltration of tissues, the predominant characteristic figure, in most of the cases, the outnumbered T cells and in other cases B cells. The lateral situation becomes apparent with the presence of certain autoantibodies first Ro/SSA and La/SSB and second rheumatoid factor, anti-fodrin antibodies, etc.

Despite the fact that the pathophysiology of SS has been intensively studied it is not completely elucidated. It is well established that genetic predisposition and different environmental or hormonal factors may well contribute and promote deregulation of the glandural epithelial cells. These potential triggers lead to the release of adhesion molecules and chemokines like CD54/ICAM-1 and CD40 from the epithelial cells (7). These molecules recruit plasmacytoid dendritic cells and T-lymphocytes to the glands. Dendritic cells release high levels of IFN-a which perpetuates the recruitment and retention of the T-lymphocytes, mainly CD4+ resulting in the glandular infiltration of the glands, one of the highlights of the SS. The activated T-cells produce various cytokines, namely, IFN-γ, IL-2, IL-6, IL-10, etc., that are correlated to the lesions of SS (8).

At the same time, interferons promote the secretion of B-cell activating factor (BAFF) from the epithelial, dendritic, and T cells. It is well depicted in certain studies an augmentation in BAFF’s serum levels of SS patients which is correlated to the deregulation of B cells. This situation has as a result the presence of autoantibodies like anti-SSA/Ro, anti-SSB/La, etc. (9, 10). Genetic predisposition according to researchers implicates the major histocompatibility complex class II gene region, especially HLA-DR and HLA-DQ alleles. Most of the related studies are devoted to specific genes for proteins related to innate and adaptive immunity like PT-PN22W variant (11) or TNFAIP3 variant (12) trying to prove the genetic background of SS. In addition, a lot of interest is accumulated toward different epigenetic factors as miRNA, long non-coding RNA, and DNA methylation (13–15).

It is generally accepted that genetics in tandem with environmental factors, especially viruses (chronic hepatitis virus, Epstein–Barr virus, etc.), provokes the initiation of a cataract that leads to inflammation and autoimmunity with glandural and extra glandural manifestations (16).

Recent research conducted SS in regard to symptoms and diagnosis are focused upon the discrimination of different subtypes of pSS (17), the evaluation of independent risk factors for lymphoma development (e.g., RF positivity, Raynaud’s phenomenon) (18), neurological and renal involvement and comorbidities (e.g., cardiovascular disorders, depression, etc.) (17).

As regards treatment options the truth is that scientists walk on the dark side of the moon. In the dawn of 2017, Birt et al. in a population-based study which included more than 10,000 patients with pSS, suggested that first line drugs are the symptomatic and immunosuppressive ones whereas there is a lack of biologic therapies prescriptions (19). According to other studies potential therapeutic interventions include anti-BAFF antibodies or BAFF antagonists (e.g., Belimumab, Atacicept, Briobacept, etc.). In addition, anti-CD20 and anti-CD22 therapy results to B-cell depletion leading to improvement in SS symptoms (e.g., rituximab), methotrexate as an immunosuppressive drug as well and recently interleukin targeted treatment (e.g., IL-6 tocilizumab) (16, 20–26). Unfortunately, all these options still require human trials to prove their effectiveness and especially in pSS patients.

## A New Actor on Stage: The Human Microbiome

During the last decades, cutting-edge together with the expansion of bioinformatics facilitated the scientists to elucidate the commensal, symbiotic and pathogenic microbial communities of the human body. The next generation sequencing (NGS) methodologies encompass the 16S ribosomal RNA (r RNA) gene sequencing and metagenomic shotgun sequencing (27). The Human Microbiome Project from the National Institute of Health in the United States was the most famous initiative that characterized the bacterial composition in different body sites (28, 29) and specifically the abundance, diversity, and the features of all the microorganisms’ genes. In fact, we are more microbes than humans as the proportion of the microbe cells to human ones is 10 to 1. The human microbiome is consisted of 100 trillion bacteria, protozoa, fungi, and viruses. It is of paramount importance contributing to health and disease through numerous biological procedures as energy extraction, regulation of metabolism, protection from pathogenic microbes, production of vitamins and last but not least regulation of our immune system (28, 29). The most abundant site of the body is the gut with 500–1,000 species. Most of the microbes belong to four major phyla: Firmicutes, Bacteroidetes, Actinobacteria, and Proteobacteria (30–32) and actually Bacteroidetes along with Firmicutes represent more than 90% of the whole in the gut (31, 32). Presumably, due to its numerous microbiota and significant metabolic activity, the human intestine is described as an “active organ” (33). Data from 16S RNA gene and whole genome shotgun succeeded to classify Europeans to three enterotypes with regard to variation of gut microbiome (31, 34).

There several factors which affect the numbers, composition, and diversity of intestine microbiota from newborn till the elderly such as gender, diet habits, smoking, vaccinations, infections, age, stress, etc. During the first week of its life, the newborn develops an augmented number of gut bacteria that as it seems depends mainly not upon delivery mode but upon mode of feeding. Breast-feeding offers greater counts of bacteria comparing to more solid food, whereas cesarean versus vaginal delivery mode shows similar bacteria counts but from different origin (mother’s vagina versus skin origin) (33, 35). Survey studies depict that diversity and richness augment during life up to adulthood and then gradually reduce (36). A healthy human intestine microbiome is characterized by high diversity while any kind of loss of diversity may lead to dysbiosis, a critical condition of disproportion between commensal and pathogenic bacteria. These commensals fill an important niche with their up or down regulation of immune response and by maintaining a homeostatic environment. As a result of these observations, there are numerous publications for the possible interaction between dysbiosis and inflammatory and metabolic diseases, for instance, obesity, cancer, asthma, and autoimmune diseases as inflammatory bowel disease (IBD), Crohn’s disease, RA, etc. (36). Intriguingly, these studies have failed up to now to establish a causative role for the microbiome but mainly focus upon the interplay between pathogenesis, clinical manifestations, and disease prognosis with the microbiome alterations.

Host immunity to pathogens is largely regulated by the commensal microbiota as numerous studies have shown. On the other hand, the microbiota implication in autoimmune response’s regulation is still a matter of discussion and research (37). Recent findings describe the effect of microbiome shifts toward the emergence of autoimmunity. The introduction of high-throughput methods in microbiome analysis like sequencing of 16S rRNA, next-generation sequencing (NGS), and metabolomics offered the opportunity for large cohort studies which revealed that the role of microbiota in the context of autoimmunity could be “protective, neutral, or provocative” as Yurkovetskiy et al. pointed out (38). Microbiota may have impact on autoimmunity and autoimmune disease development in different means. Direct effects of commensals include the well established molecular mimicry as potential ligand to autoimmunity through the existence of non-selective T cell receptors with cross-reactivity to self-antigens. In addition, many innate immune sensors are activated by microbe components (pathogen-associated molecular patterns), and microbial metabolites might be another part of a chain in this procedure (38). Diet interventions could affect target organs like pancreas or neural tissue by microbiota shifts or in a straight pathway. Relevant experiments in NOD mice demonstrated that diet changes decreased the incidence of T1D on the if only started from the utero life (39). Sexual dimorphism, a prominent characteristic of many autoimmune diseases, according to recent studies, may be attributed to a microbiota role in hormonal alterations (40, 41).

All these interactions are sufficiently complicated and future research in gender-specific microbiota and its relations in tandem with cautious explanation of experimental results will probably enlighten the dark sides of the autoimmune conditions.

## Bidirectional Relation Between Microbiome and SS

Having considered all the above it is more than obvious the accumulating research currently focuses on the contribution of human microbiome to systemic autoimmune diseases (SADs). Bacteria of the microbiome get in touch with the mucosal immune system and after various interactions can lead to dysbiosis with local inflammation and disruption of gut barrier. As a cataract numerous consequences might occur, for instance, pro-inflammatory cytokines in systemic circulation, distant impact of inflammation such as joints, greater antigen exposure with the increase of autoantibody production, etc. SADs are multifactorial diseases implicating genetic predisposition, environmental factors, hormonal influences, and a disordered immune system (42).

Studies have suggested that T and B cells in the mucosa significantly interfere and promote the immune homeostasis working in two different fields, harnessing of immune response against beneficial microbes and maintaining the wholeness of gut barrier (43). Some bacteria from the microbiota communities present unique abilities to affect and foster the activation and polarization of certain lymphocyte subsets. For instance, experiments in mice revealed that T helper 17 (Th17) are favored by segmented filamentous bacteria in small intestine resulting in autoimmune arthritis (44, 45). But Th17 cells’ functionality may present two sides of the same coinas they may act either by preventing infection or be pathogenic by secreting pro-inflammatory cytokines (46–49). It is still vague the exact mechanism that governs this procedure of Th differentiation.

Another lymphocyte subpopulation that comprise a large number is the T regulatory (Treg) cells. Tregs are invaluable for their contribution in immune tolerance to diet-origin antigens and gut microbes. Studies concerning the magnitude of influence of intestine microbiota to the induction of Tregs are still in their infancy. For example, Tregs are induced by *Clostridium* species in intestine with anti-inflammatory performance (50, 51).

**Figure 1:**
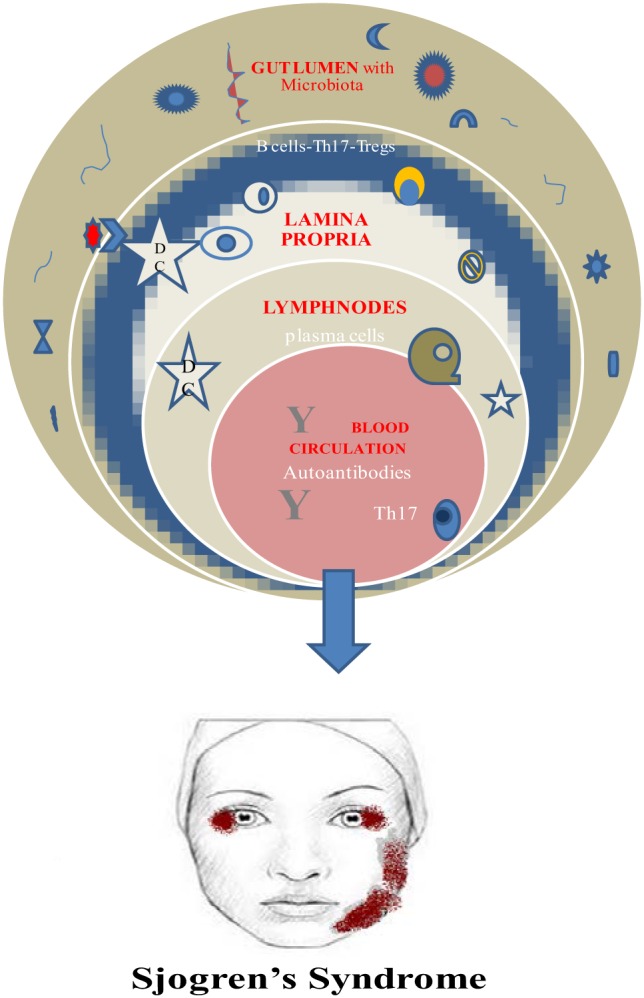
Microbiome and Sjogren’s syndrome.

Finally, a vital requirement for this beneficial role of microbiota is its establishment early in life. Otherwise, the prevalence of invariant natural killer cells and the suppression of Tregs are connected to colitis and asthma in mice (52, 53) (Figure 1).

Genetic background and environmental factors have not gain much attention especially for SS patients despite the fact that scientists prove and believe in hereditary and familial predisposition and EBV association for the disease onset along with increased production of type I IFN (54–56). It is quite predictable the fact that the main body of the related studies refers to the saliva alterations due to the attack of the salivary glands. As early as 2003, Almståhl et al. observed in SS patients’ saliva an augmentation of *Candida species* and *Streptococcus mutans* whereas *Fusobacterium nucleatum* colony forming units were depleted (57). Moreover, de Paiva et al. found additionally high levels of *Lactobacillus* spp. in supragingival plaque samples, *Staphylococcus aureus* along with *Candida albicans* in oral mucosa and tongue, and a decrease in *Leptotrichia* and *Fusobacterium* (6). Concerning the gut microbiota researchers revealed depletion of *Faecalibacterium, Bacteroides, Parabacteroides*, and *Prevotella* and augmentation of *Escherichia, Shigella*, and *Streptococcus genera* (6). It seems that only Szymula et al. managed to set the hypothesis in a transgenic murine model that dendritic cells with a microbial protein like von Willebrand factor type A (or other peptides produced from human commensals) could activate T cells with a Ro60 receptor and leads to autoantibody production (58). Molecular mimicry as a possible mechanism for autoimmunity could explain the microbiome–SS connection (58), and deregulated immune response fighting the normal microbiome could be considered as a potential pathway in SS pathogenesis and disease perpetuation. Regretfully, just hints and indirect evidences are the only elements in this puzzle up to now.

It is generally accepted the pathophysiological role of autoreactive B cells and Th17 cells in SS and the direct or indirect implication of the human microbiome.

Th17 cells are present in salivary glands of SS patients and in peripheral blood as well. So it could be a solid hypothesis the increase of this population due to dysbiosis leads to entering the circulation and reach the exocrine glands. Unfortunately, it is still unclear if the origin of the Th17 cells is lamina propria of the gut and the pattern that gut immunity triggers autoimmune procedures at distal sites (i.e., salivary glands) (42).

## Microbiome Shifts and Future Dietary Interventions in SS

Collectively, all the above information has stressed on the “fight” against dysbiosis which is clearly implicated in the onset and continuing of autoimmunity. Different studies on SS have presented indirect evidence in shifts of oral, skin, and gut microbiome (58, 59) but not direct connection leading to the hypothesis that if the microbiome really plays an important role then novel treatment ways such as diet interventions and “functional food” could offer an alternative to traditional immunosuppressive therapies. Dysbiosis as a condition of aberrant function of microbiota that leads to deregulation of immune and metabolic homeostasis, low-grade chronic inflammation may contribute or predispose to a wide range of inflammatory diseases such as allergy, asthma, autoimmune diseases, obesity and metabolic disorders, cognitive and mental health dysfunction, etc. (60). Targeting dysbiosis by implementing diet-induced shifts in microbiome may affect the development of autoimmunity. Efforts to correct the malfunction of the disturbed gut–barrier encompass generally probiotics, prebiotics, dietary fiber, and fecal microbiota transplantation depending on the disease.

Probiotics are live microorganisms which when administered in adequate amounts confer a health benefit on the host (61). Probiotic microorganisms include principally bacteria that produce lactic acid as *Lactobacillus* and *Bifidobacterium* genera (LAB). Their use is mainly to restore the microbiota imbalance by different mechanisms that are still under research (62) even though there are plenty of studies that demonstrate the beneficial effect on allergic diseases (e.g., asthma and eczema), obesity, metabolic syndrome, gastrointestinal disorders, etc. (60). Prebiotics are non-digestible fermentable oligosaccharides that could alter the composition and/or the functionality of the gut flora and contribute to the increase of the beneficial bacteria, namely, *Lactobacilli* and *Bifidobacteria* (36, 63, 64). In the same manner, a combination of pre- and probiotics (symbiotic) has been used (65).

Currently, researchers spark interest in the therapeutic capability of probiotics and prebiotics based approaches regarding autoimmune diseases. This strategy might represent a more natural way to master the autoimmune response by avoiding any side effects of immunosuppressive drugs. The most recent studies focus on the effect of functional food (prebiotics and probiotics) on Treg cells concerning autoimmune disorders (66). Natural Treg cells population represents the cornerstone for the peripheral tolerance, playing a crucial role to suppress the autoreactive T-cells which have surpassed thymus supervision and cancel any autoimmune response. Any kind of inadequacy of these cells as regards the number or activity seems to be correlated with different autoimmune disorders, for instance, dermatomyositis (67), systemic lupus erythematosus (68), or vitiligo (69). Konieczna et al. in 2012 revealed that certain probiotics can promote the induction of Tregs in patients with inflammatory disease (70). This induction could restore the imbalance between Tregs and effector CD8+ CD4+ Tcells (in greater number in autoimmunity) resulting in immune homeostasis (decrease of pro-inflammatory factors, augmentation of anti-inflammatory factors, increase in number and activity of Tregs, decrease of cytotoxicity, etc.) (69). In a similar manner, prebiotics and their metabolites in recent reports seem to compel to homeostasis by different mechanisms such as short chain fatty acids induction of Tregs, by prostaglandin E2, etc. (69).

Indeed, there is a bulk of evidence that there are certain bacteria which could affect the induction of Treg cells in the intestine and prevent Th17 expansion like *Bacteroides fragilis* and *Clostridium* strains in experimental colitis, T1DM and IBD. These results could be valuable lessons for future studies in animal and humans regarding SS and the shaping of the microbiota to suppress autoimmunity. Given the crucial role of the benefic bacteria and the possible diet interventions using probiotics and prebiotics, SS patients could ameliorate disease severity and prediction. The paucity of studies concerning specifically this entity, either animal or human studies reveals the magnitude of the problem. Knowledge from other autoimmune disorders could facilitate the assumption that microbiome manipulation may alleviate the disease burden.

## Conclusion

Sjogren’s syndrome as a common, multifactorial autoimmune disorder remains till now mostly underdiagnosed and undertreated. At the same time as the latest breakthroughs in culture-independent techniques of studying human microbiome revealed its possible connection with SS pathogenesis, onset, severity, and treatment, scientific community sparked off a new field of interest. Having considered pioneer research as regards autoimmunity and microbiota in general and the profound contribution of dysbiosis, future efforts possibly would be focused first, on the determination whether the reduced/altered microbial diversity is causal. Second, it seems essential to define the precise mechanisms of microbiome shifts’ occurrence driving by specific bacteria as well the exact subpopulation of the patients that potential manipulations would be truly auspicious complied with personalized medicine. The intricate knowledge of human microbiota with the positive, negative and neutral bacteria in tandem with their resilience and stability (71) on one hand and specific markers for the efficiency of the interventions on the other, would give prominence and real value to the outcome of a microbiome-centered treatment strategy. Functional food, namely, prebiotics and probiotics offer a very promising future concerning SS therapies with diet interventions and may prove to be the ultimate immunomodulatory factors to suppress autoimmunity. The very encouraging results of Zamani et al. in 2016 (72) who demonstrated the beneficial effects of 8 weeks probiotic supplementation in RA patients on their clinical and metabolic status, might be only the start of personalized manipulations for SS and other autoimmune entities, to shift their microbiome from disease to health.

## Author Contributions

EB supervised the work. CT was responsible for the laboratory data and part of the work and manuscript. ES was responsible for the clinical aspects of this review.

## Conflict of Interest Statement

The authors declare that the research was conducted in the absence of any commercial or financial relationships that could be construed as a potential conflict of interest.
